# Exploration of risk factors for the occurrence and recurrence of papillary thyroid carcinoma with Hashimoto's thyroiditis based on next-generation sequencing

**DOI:** 10.1186/s13000-025-01639-w

**Published:** 2025-04-23

**Authors:** Wanxue Zhang, Bin Liu

**Affiliations:** Department of General Thyroid Surgery, The First Affiliated Hospital of Anhui Medical University, Hefei, 230031 China

**Keywords:** Papillary thyroid carcinoma, Hashimoto's Disease, Next Generation Sequencing, RET rearrangement

## Abstract

**Objectives:**

To investigate the risk factors for the occurrence and recurrence of Hashimoto's thyroiditis (HT) combined with papillary thyroid carcinoma (PTC) using Next-Generation Sequencing (NGS).

**Methods:**

A total of 249 patients who underwent thyroid cancer surgery at the First Affiliated Hospital of Anhui Medical University from October 2020 to December 2020 were included in this study. They were divided into two groups: HT and non-HT (NHT) groups based on whether they were diagnosed with HT or not. Clinical data, NGS results, and 4-year follow-up recurrence were collected and analyzed using binary logistic regression and COX regression analysis to identify factors influencing the occurrence and recurrence of PTC with HT.

**Results:**

Patients in the HT group had a higher percentage of low age, multifocality, high TgAb, and RET rearrangement compared to the NHT group. However, they had a lower rate of extrathyroidal extension (ETE), lymph node metastasis (LNM), and BRAF mutation (*P* < 0.05). Among patients with RET rearrangement in the HT group, there was a higher rate of LNM and recurrence (*P* < 0.05). Patients with BRAF mutation in the HT group were more likely to be male and have multifocal tumors (*P* < 0.05). Binary logistic regression analysis showed that multifocality, RET rearrangement, low age, and lymph node negativity were risk factors for HT combined with PTC. Univariate COX analysis revealed that multifocality, LNM, ETE, recurrence risk stratification, TSH, RET rearrangement, and the interaction between RET rearrangement and Hashimoto's effect were risk factors for recurrence after PTC. Multifactorial COX regression analysis showed that ETE and recurrence risk stratification were risk factors for recurrence after PTC surgery.

**Conclusions:**

Multifocality, high TgAb, low age, and lymph node negativity can assist in the preoperative diagnosis of HT combined with PTC. BRAF mutations are less common in HT combined with PTC but do not significantly affect recurrence. Patients with RET rearrangement in addition to HT have a higher risk of recurrence, and special attention should be paid to intraoperative clearance in these patients.

## Introduction

Hashimoto's thyroiditis (HT) is an autoimmune inflammatory disease of the thyroid gland and the leading cause of hypothyroidism. Papillary Thyroid Carcinoma (PTC) is the most prevalent subtype of thyroid cancer and has a favorable prognosis. However, it can also present early with cervical LNM, and distant metastasis can occur in later stages of disease progression. Since Dailey et al. [1] first found an association between HT and PTC in 1955, more in-depth studies have been carried out. These studies suggest that HT and PTC may have a better clinical prognosis [2]. However, there are still some articles [3] pointing out that HT combined with PTC has a higher malignancy and LNM rate. Other studies [4] have found that PTC combined with HT may have a centralized regional neck lymph node metastasis with a lower probability, but it is a risk factor for cervical lateral lymph node metastasis (LLNM). The pathogenesis of HT combined with PTC is still in the exploratory stage, and it is unclear whether the relationship between the two is causal or incidental [5–7]. Additionally, the reactive hyperplasia of lymph nodes induced by HT [8] increases the difficulty of preoperative assessment of LNM and intraoperative lymph node clearance. This may lead to residual cancer and a higher risk of recurrence. Postoperative complications, such as postoperative hypocalcemia and hoarseness [9], can also increase the probability of recurrent secondary surgery due to scar proliferation and tissue adhesions from previous neck surgery. The guidelines have not yet provided clear guidance on the extent of surgery for patients with PTC combined with HT. This is especially true for whether unilateral total excision is necessary and whether lymph node dissection should be performed for those with suspicious cervical lymph node enlargement suggested by preoperative imaging data. Furthermore, many studies have shown that HT is often combined with microscopic and occult cancers [10–13], which can lead to missed diagnosis and missed resection. Therefore, postoperative pathologic diagnosis should be assisted by a combination of comprehensive genetic testing. The presence of a dense lymphocytic infiltration in HT may reduce the sensitivity of methods for detecting relevant mutations in thyroid cytology specimens [14, 15]. Next-Generation Sequencing (NGS), a powerful tool for detecting mutations in thyroid cytology specimens, is a next-generation sequencing technology with high throughput, high depth, and high sensitivity. It has been widely used in basic or clinical studies of various cancers [16]. The aim of this study was to use NGS technology to detect relevant gene mutations, explore the risk factors for the occurrence and recurrence of HT combined with PTC, and provide more references for the development of clinical surgical protocols.

## Materials and methods

### General information

This study is a retrospective cohort study that consecutively included 249 patients who underwent thyroid cancer surgery from October 2020 to December 2020 in the First Affiliated Hospital of Anhui Medical University. Clinical data including age, gender, thyroid function, tumor diameter, postoperative pathology with or without ETE number of cancer foci, and presence of LNM were collected from the patients.

Inclusion criteria: ① all met the clinical diagnostic criteria of PTC and HT and were confirmed by postoperative pathological examination; ② all received surgery and other related treatments for the first time; ③ all did not have the combination of cardiovascular, cerebral vascular, hepatic, renal, and other important organs and tissues diseases; ④ all voluntarily participated in the study and signed the informed consent.

Exclusion criteria: ① Combined with other thyroid diseases such as primary hyperthyroidism, positive thyroid stimulating hormone receptor antibody or diffuse enlargement of the thyroid gland; ② Previous thyroid surgery or combined with hyperthyroidism; ③ Combined with follicular or medullary carcinoma and other types of thyroid cancer; ④ Combined with immune, hematological diseases or other malignant tumors; ⑤ Clinical information is incomplete or the degree of cooperation of the patients is poor.

Diagnostic criteria: Diagnostic criteria of HT: thyroid tissue is diffusely enlarged and hard in consistency; preoperative ultrasound shows that the thyroid gland is diffusely echogenic; preoperative thyroid function shows that thyroid peroxidase antibody (TPOAb) and thyroglobulin antibody (TgAb) are elevated; and pathological findings suggest HT. Diagnostic criteria for thyroid cancer: preoperative puncture pathology or postoperative pathology suggestive of thyroid cancer.TNM staging was based on the staging definition criteria in the eighth edition of the American Joint Committee on Cancer (AJCC).

All patients signed an informed consent form, and the study was approved by the Ethics Committee of the First Affiliated Hospital of Anhui Medical University.

### Tumor tissue gene sequencing

Fresh tissue specimens obtained from surgery were immediately frozen in liquid nitrogen and stored at − 70 °C until sequencing. DNA was extracted using HiPure FFPE DNA kit and RNA was extracted using RNApure FFPE kit, and the extracted DNA and RNA samples were used for library construction, and NGS technology was applied for nucleic acid sequencing, and the data were filtered and compared with the nucleic acid sequences in the libraries. We analyzed and detected all the hotspot exon regions and some introns of human BRAF, RET, EGFR, FGFR, HRAS, KRAS, NRAS, NTRK, TERT and other genes.

### Postoperative follow-up

After surgery, all patients took levothyroxine tablets for long-term TSH suppression therapy, and none of them underwent chemotherapy or radiation therapy after surgery. The patients were followed up at regular intervals, every 1 month for the first 3 months, every 3 months for the first year, and every 6 months thereafter. Follow-up visits were mainly in outpatient clinics and by telephone, and included thyroid function and thyroid ultrasound. Patients who did not have regular follow-up and did not take medication regularly were regarded as lost patients, and if the follow-up patient was diagnosed with recurrent/persistent thyroid cancer, the follow-up would end.

Diagnostic criteria for recurrent/persistent thyroid cancer [17]: reappearance of biochemical or imaging abnormalities after initial treatment after negative imaging performance and failure to measure thyroglobulin (Tg) on stimulation with TSH, and the site of recurrence can be a remnant gland, or extra-glandular in the form of lymph node metastasis or distant metastasis.

### Statistical analysis

This study used SPSS version 25.0 to statistically analyze the test results and clinicopathological characteristics. Categorical variables were analyzed using the chi-square test or Fisher's exact test, and expressed as the number of cases and percentage (%); continuous variables were analyzed using the t-test or Mann–Whitney U-test, and normal independent samples were described by mean ± standard deviation, and non-normal measures were described by quartiles M (Q1-Q3); the factors affecting the occurrence of HT combined with PTC were analyzed using binary logistic regression; Cox regression was used to analyze the factors affecting recurrence; *P* < 0.05 was statistically different from each other. analysis; Cox regression method was used to analyze the factors affecting recurrence; *P* < 0.05 was statistically different.

## Results

### Comparison of clinical features, thyroid function indexes and NGS results between the HT and NHT groups

In the NGS results, gene mutations were detected in 195 patients (78.31%) out of 249 PTC specimens. Among these 249 patients, 159 patients (63.86%) had single gene mutations and 36 patients (14.46%) had 2 or more gene mutations. Among the detected genes, BRAF gene had the highest mutation rate (164 cases) with a mutation rate of 65.86%, and the rest were, in order, RET (20 cases), TERT (13 cases), NRAS/HRAS, TP53 mutation (5 cases), TSHR (3 cases), and PIK3 CA (3 cases) (the remaining mutated genes were less than or equal to 1 case were not discussed).

**Fig. 1:**
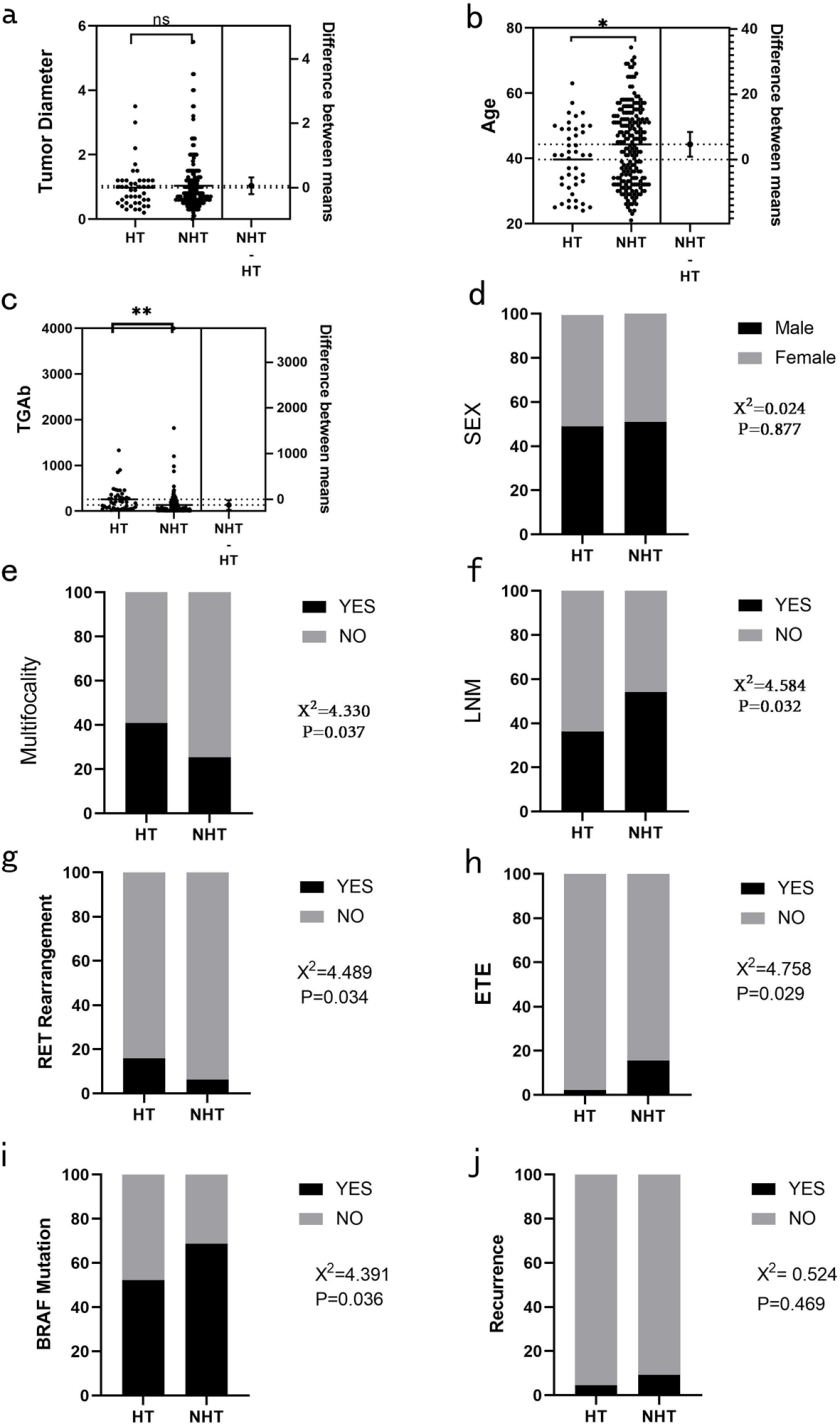
(a-j) Comparison of basic information between the Hashimoto group and the control group

Compared with the NHT group, patients in the HT group were younger, had higher TgAb, less ETE, more multifocality, lower LNM rate, lower BRAF mutation rate, and higher RET rearrangement rate (*P* < 0.05). There were no statistical differences between the two groups in gender composition, maximum tumor diameter, recurrence rate, TERT mutation, NRAS/HRAS mutation, TSHR mutation, TP53 mutation, PIK3 CA mutation, and NTRK1 mutation (*P* > 0.05). (Fig. 1, Table 1).

**Table 1:** Comparison of basic information between HT group and NHT group

Characteristics	HT (*n* = 44)	NHT (*n* = 205)	t/Z (X^2^)	*P*-value
Age	41 (31.25,49)	45 (33.5,53)	2.431	0.016
Sex			(0.024)	0.877
Male	13 (29.55%)	63 (30.73%)		
Female	31 (70.45%)	142 (69.27%)		
ETE			(4.758)	0.029
Yes	1 (2.27%)	32 (15.61%)		
No	43 (97.73%)	173 (84.39%)		
Tumor Diameter (cm)	0.95 (0.5,1.2)	0.8 (0.5,1.2)	0.457	0.648
Multifocality			(4.330)	0.037
No	26 (59.09%)	153 (74.63%)		
Yes	18 (40.91%)	52 (25.37%)		
LNM			(4.584)	0.032
No	28 (63.64%)	94 (45.85%)		
Yes	16 (36.36%)	111 (54.15%)		
TGAb	269 (61.95,417)	14.73 (4.98,28.07)	-	<0.001
BRAF Mutation			(4.391)	0.036
Yes	23 (52.27%)	141 (68.78%)		
No	21 (47.73%)	64 (31.22%)		
RET rearrangement			(4.489)	0.034
Yes	7 (15.91%)	13 (6.34%)		
No	37 (84.09%)	192 (93.66%)		
TERT Mutation			(<0.001)	1.000
Yes	2 (4.55%)	11 (5.37%)		
No	42 (95.45%)	194 (94.63%)		
NRAS/HRAS Mutation				0.594
Yes	0 (0.00%)	6 (2.93%)		
No	44 (100.00%)	199 (97.07%)		
TSHR Mutation			(<0.001)	1.000
Yes	1 (2.27%)	2 (0.98%)		
No	43 (97.73%)	203 (99.02%)		
TP53 Mutation				0.590
Yes	0 (0.00%)	5 (2.44%)		
No	44 (100.00%)	200 (97.56%)		
PIK3CA Mutation			(2.812)	0.140
Yes	2 (4.55%)	1 (0.49%)		
No	42 (95.45%)	204 (99.51%)		
Recurrence			0.524	0.469
Yes	2 (4.55%)	19 (9.27%)		
No	42 (95.45%)	186 (90.73%)		

*ETE* Extrathyroidal extension, *LNM* Lymph node metastasis

### Comparison of clinical characteristics of patients with RET rearrangements and BRAF mutations in the HT group

2.2.1 Compare the clinical characteristics of patients with positive and negative RET rearrangement in the HT group. Patients with RET rearrangement had a higher incidence of LNM and LLNM and a higher recurrence rate (*P* < 0.05), while all other clinical characteristics were not statistically significantly different from those of the NRET group (Table 2).

**Table 2:** Comparison of basic information between RET rearrangement-positive and negative groups in the HT group

Characteristics	RET Rearrangement		t/Z（*X*^2^）	*P*-value
	Positive (*n* = 7)	Negative (*n* = 37)		
Age	34 (25,50)	41 (32,49)	-0.627	0.531
Sex			(<0.001)	1.000
Male	2 (28.57%)	11 (29.73%)		
Female	5 (71.43%)	26 (70.27%)		
ETE				0.159
Yes	1 (14.29%)	0 (0.00%)		
No	6 (85.71%)	37 (100.00%)		
Tumor Diameter (cm)	1 (0.6,3)	0.9 (0.5,1.2)	-0.903	0.377
Multifocality			(<0.001)	1.000
No	4 (57.14%)	22 (59.46%)		
Yes	3 (42.86%)	15 (40.54%)		
LNM				<0.001
No	0 (0.00%)	28 (75.68%)		
Yes	7 (100.00%)	9 (24.32%)		
LLNM			(5.750)	0.016
No	5 (71.43%)	30 (81.08%)		
Yes	2 (28.57%)	7 (18.92%)		
Recurrence				0.022
Yes	2 (28.57%)	0 (0.00%)		
No	5 (71.43%)	37 (100.00%)		

*ETE* Extrathyroidal extension, *LNM* Lymph node metastasis, *LLNM* Lateral neck lymph node metastasis

2.2.2 Compare the clinical characteristics of BRAF mutation positive and negative patients in the HT group. Patients who developed BRAF mutations were more male and multifocal (*P* < 0.05), and other clinical features and the presence or absence of LNM and recurrence rate were not statistically significantly different from those of the NBRAF group (Table 3).

**Table 3:** Comparison of basic information between BRAF mutation positive and negative groups in HT group

Characteristics	BRAF Mutation		t/Z（*X*^2^）	*P*-value
	Positive (*n* = 23）	Negative (*n* = 21)		
Age	37 (27,47)	42 (34,50)	-1.558	0.112
Sex			(6.006)	0.014
Male	11 (47.83%)	2 (9.52%)		
Female	12 (52.17%)	19 (90.48%)		
ETE				1.000
Yes	1 (4.35%)	0 (0.00%)		
No	22 (95.65%)	21 (100.00%)		
Tumor Diameter (cm)	1 (0.4,1.2)	0.9 (0.55,1.2)	-0.756	0.450
Multifocality			(4.859)	0.027
No	10 (43.47%)	16 (76.19%)		
Yes	13 (56.52%)	5 (23.80%)		
LNM			(2.736)	0.098
No	12 (52.17%)	16 (76.19%)		
Yes	11 (47.83%)	5 (23.80%)		
LLNM			(0.243)	0.622
No	16 (69.57%)	16 (76.19%)		
Yes	7 (30.43%)	5 (23.80%)		
Recurrence			(<0.001)	1.000
Yes	1 (4.35%)	1 (4.76%)		
No	22 (95.65%)	20 (95.24%)		

*ETE* Extrathyroidal extension, *LNM* Lymph node metastasis, *LLNM* Lateral neck lymph node metastasis

### Binary logistic regression analysis of independent risk factors causing HT combined with PTC

**Fig. 2:**
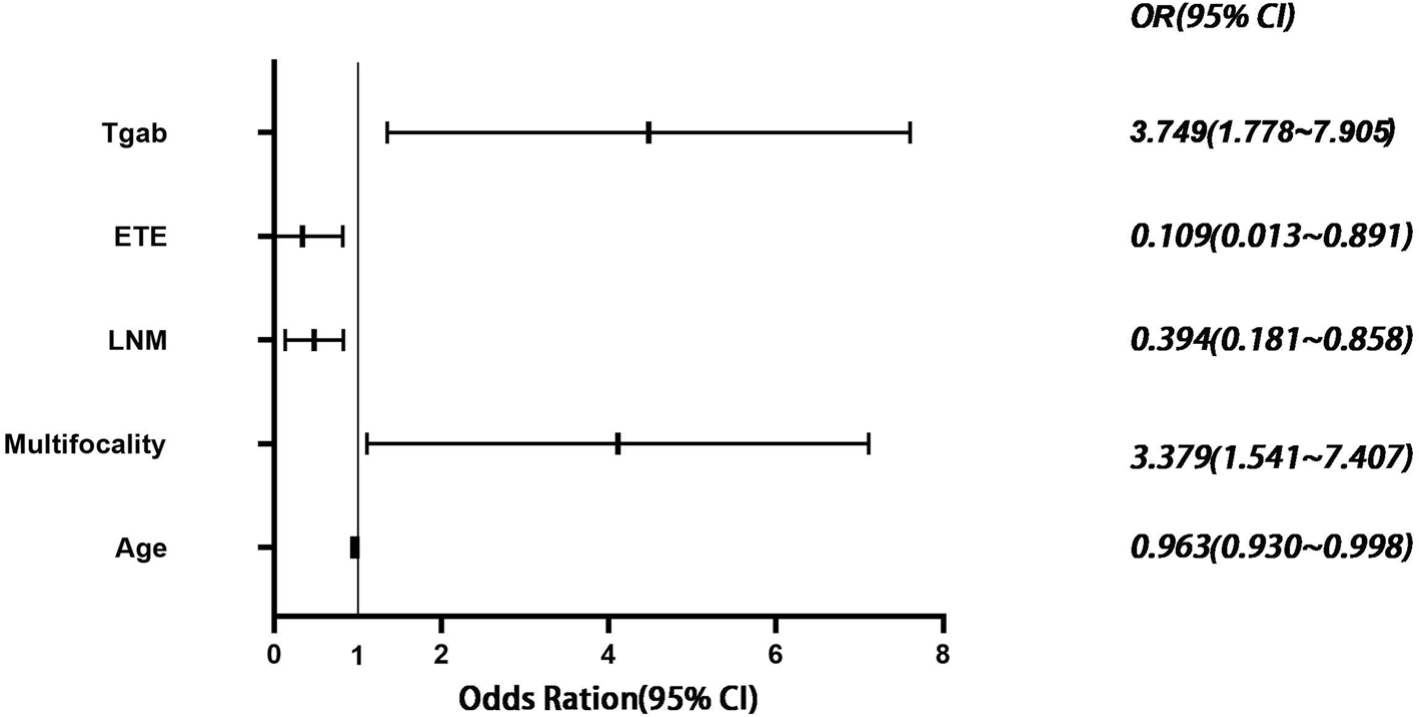
Binary Logistic Regression Analysis of Independent Risk Factors Causing HT Combined with PTC

According to the assignment table (Table 4), whether combined HT was the dependent variable, age, whether multifocal, whether LNM, whether ETE, TgAb, BRAF mutation, and RET rearrangement were used as the independent variables, and the analysis of regression model showed that multifocal and RET rearrangement were the significant positive influences of HT combined with PTC, and advanced age and LNM had a significant negative influence on them, and ETE, TgAb, and BRAF mutation did not constitute a statistically significant effect on it. (Fig. 2).

**Table 4:** Multi-factor assignment table

Variant	Assignment
ETE	Yes=1, No=0
LNM	Yes=1, No=0
Multifocality	Yes=1, No=0
BRAF Mutation	Yes=1, No=0
RET Rearrangement	Yes=1, No=0

*ETE* Extrathyroidal extension, *LNM* Lymph node metastasis

### Analysis of recurrence

All 249 patients were effectively followed up, and there were no lost patients, with a follow-up rate of 100.00%. The follow-up was for four years with a cut-off date of October 2024, and the median follow-up time was 38.1 months. Overall recurrent/persistent thyroid cancer occurred in 21 cases with a recurrence/persistence rate of 8.43%. The initial surgical approach and site of recurrence are shown in Table 5.

**Table 5:** Initial surgical approach and recurrence site

Initial Surgical Approach	Cases	Postoperative Recurrence Cases	Recurrence Site		
			Residual Thyroid	Lymph nodes Of zone VI	lymph nodes of Lateral cervical
Unilateral thyroidectomy+ lymph node dissection in zone VI	76	7	6	3	2
Unilateral thyroidectomy+ lymph node dissection in Unilateral Lateral cervical	11	-	-	-	-
Total thyroidectomy+ lymph node dissection in zone VI	117	14	-	3	14
Total thyroidectomy+ lymph node dissection in Unilateral Lateral cervical	41	-	-	-	-
Total thyroidectomy+ lymph node dissection in Bilateral Lateral cervica	4	-	-	-	-

Log-rank test showed that the overall recurrence rate was 4.55% (2/44) in the HT group and 9.27% (19/205) in the NHT group, and the difference was not statistically significant (X^2^ = 0.772,*P* = 0.380); the BRAF mutation-positive recurrence rate was 4.35% (1/23) in the HT group, and the overall recurrence rate was 4.76% (1/21) in the NHT group. The difference was not statistically significant (X^2^ = 0.007,*P* = 0.935); whereas the positive recurrence rate of RET rearrangement in the HT group was 28.57% (2/7), and the total recurrence rate in the NHT group was 0% (0/37), and the difference was statistically significant (X^2^ = 11.403,*P* = 0.001);

### Unifactorial and multifactorial COX regression analysis of independent risk factors causing HT combined with PTC

2.5.1 Unifactorial cox regression analysis of the influencing factors of recurrence in patients in the HT group suggested that tumor diameter and ETE (*P* < 0.05) were the correlates of postoperative recurrence in the HT group, whereas gender, TSH, age, the number of cancerous foci, LNM, BRAF mutation, and RET rearrangement had nothing to do with them. (Table 6).

**Table 6:** Univariate cox regression analysis of factors influencing recurrence in patients in the HT group

Characteristics	β	SE	Wald	P	HR	HR (95% CI)
Age	0.853	1.414	0.363	0.547	2.346	0.147~37.508
Sex	0.114	0.081	1.997	0.158	1.121	0.957~1.314
Tumor Diameter (cm)	1.626	0.700	5.395	0.020	5.083	1.289~20.043
Multifocal	4.651	6.017	0.597	0.440	104.658	0.001~13841512.980
LNM	4.953	6.337	0.611	0.434	141.601	0.001~35098537.790
ETE	3.749	1.414	7.029	0.008	42.497	2.658~679.455
Recurrence risk Stage	6.633	10.338	0.412	0.521	759.836	0.000~4.787E+11
TSH	-1.294	0.924	1.962	0.161	0.274	0.045~1.677
BRAF Mutation	-0.115	1.414	0.007	0.935	0.891	0.056~14.248
RET Rearrangement	8.712	23.055	0.143	0.706	6075.663	0.000~2.558E+23

*ETE* Extrathyroidal extension, *LNM* Lymph node metastasis

2.5.2 Univariate cox regression analysis of the influencing factors of overall patient recurrence suggested that multifocal, LNM, ETE, higher recurrence risk stage, higher TSH, positive RET rearrangement, and RET rearrangement interacting with Hashimoto's effect were the correlates of postoperative recurrence after PTC (*P* < 0.05), whereas gender, age, tumor diameter, simple combined Hashimoto's, positive BRAF mutation, BRAF-interacting RET and BRAF-interacting bridging effects were not related to them (*P* > 0.05). (Table 7).

**Table 7:** Univariate cox regression analysis of factors influencing recurrence in overall patients

Characteristics	β	SE	Wald	P	HR (95% CI)
Age	0.182	0.469	0.150	0.698	1.200 (0.478~3.009)
Sex	0.034	0.019	3.206	0.073	1.035 (0.997~1.074)
Tumor Diameter (cm)	0.134	0.255	0.274	0.601	1.143 (0.693~1.885)
Multifocal	1.615	0.469	11.858	0.001	5.027 (2.005~12.604)
LNM	1.780	0.627	8.058	0.005	5.927 (1.735~20.250)
ETE	3.159	0.518	37.248	0.000	23.554 (8.540~64.967)
Recurrence risk Stage					
1			35.364	0.000	
2	2.955	0.659	20.140	0.000	19.208 (5.284~69.826)
3	4.117	0.693	35.326	0.000	61.352 (15.786~238.448)
TSH	0.038	0.010	14.977	0.000	1.039 (1.019~1.059)
Coexistence with HT	-0.644	0.745	0.746	0.388	0.525 (0.122~2.264)
BRAF Mutation	0.691	0.560	1.523	0.217	1.995 (0.666~5.977)
RET Rearrangement	1.248	0.562	4.928	0.026	3.483 (1.157~10.484)
RET Rearrangement * Coexistence with HT	1.608	0.747	4.631	0.031	4.991 (1.154~21.585)
BRAF Mutation * Coexistence with HT	-0.659	1.026	0.412	0.521	0.518 (0.069~3.866)
BRAF Mutation * RET Rearrangement	1.755	1.032	2.893	0.089	5.783 (0.766~43.682)

*ETE* Extrathyroidal extension, *LNM* Lymph node metastasis, *HT* Hashimoto's thyroiditis

2.5.3 Multifactorial COX analysis, 6 variables of TSH, multifocal, LNM, ETE, recurrence risk stage, and RET rearrangement positivity were subjected to multifactorial COX regression analysis. The results of the analysis showed that TSH, multifocal, LNM, combined Hashimoto, and RET rearrangement had no effect on postoperative recurrence of PTC. While ETE, higher risk of recurrence stratification (*P* < 0.05) had an effect on recurrence(Table 8).

**Table 8:** Multifactorial cox regression analysis of factors influencing recurrence in overall patients

Factors	β	SE	Wald	P	HR	HR ( 95% CI)
ETE	2.003	0.597	11.265	0.001	7.410	2.301~23.864
Recurrence Risk Stage						
1	-	-	8.818	0.012	-	-
2	2.096	0.853	6.036	0.014	8.134	1.528~43.301
3	2.682	0.910	8.681	0.003	14.610	2.454~86.972

*ETE* Extrathyroidal extension

## Discussion

In the available studies [2], the prevalence of Hashimoto's thyroiditis (HT) is increased in thyroid cancer. The presence of homologous HT tends to suggest an association with less aggressive papillary thyroid cancer (PTC), such as lower TNM stage, lower likelihood of centralized LNM, and less peritumoral and vascular infiltration. This suggests a better prognosis, but the association of the coexistence of the two with recurrence and disease-free survival is not conclusive. Recurrence of thyroid cancer can include local recurrence, regional recurrence, and distant metastasis [18]. The results of this study suggest that the rate of central or lateral lymph node recurrence after thyroid surgery is much higher than that of residual thyroid body recurrence (80.95% versus 28.57%). In other words, regional lymph node recurrence predominates in the recurrence after thyroid surgery. Surgery is the first-line treatment option for thyroid cancer, both for initial diagnosis and recurrence. In our study, we found that PTC patients with combined HT were younger and had higher preoperative TgAb indexes. They also had less ETE, as suggested by postoperative pathology, and more multifocal tumors (defined as two or more anatomically independent foci in the thyroid, including unilateral or bilateral glandular lobe onset [19]. They also had lower LNM, which is consistent with previous studies [20–22] that suggest clinical features such as younger age, less ETE, and negative lymph nodes tend to suggest a better prognosis. Multifocality was also more common in PTC with combined HT in this study, which is consistent with clinical reports [23, 24]. TgAb, a clinical diagnostic indicator of HT, is considered an independent risk factor for the occurrence of LNM in HT combined with PTC [25]. This is thought to be related to the fact that the gene antigenic epitopes of thyroid carcinoma and HT can be recognized by TgAb, thus inducing tumor progression. There was no significant difference between the two groups of patients in terms of maximum tumor diameter. A comprehensive review of the literature [26] suggests that a tumor diameter of > 1 cm is a risk factor for the development of central regional LNM in PTC with combined HT, but none of the studies were able to determine a specific cut-off value for tumor diameter. HT occurs more often in women, which may be related to estrogens and progestins [27]. One study suggested that men with PTC with combined HT had a lower incidence but were more likely to develop cervical lymph node metastasis [23]. However, in this study, we did not find a statistically significant gender difference between the two groups. This may be related to a selective bias in the experimental enrollment population.

BRAF mutations are now recognized as molecular markers of poor prognosis in PTC, suggesting a poorer outcome for patients. However, in this study, BRAF mutations were not significantly associated with PTC recurrence in patients with combined HT. A previous study [8] found that BRAF mutations were present in patients with HT, but the lower rate of mutations may be linked to the relatively better prognosis of PTC patients with HT. However, another study [28] reported that BRAF mutations are a significant risk factor for LNM in PTC patients with HT. Additionally, one article [29] proposed that the PTC microenvironment is composed of a mixture of tumor cells, with only a small portion carrying BRAF mutations. This may lead to an underestimation of the frequency of BRAF mutations in PTC patients with HT, as the presence of wild-type BRAF lymphocytes can dilute the mutant cells and potentially impact the final outcome. In addition to BRAF mutations, RET chromosomal rearrangements also activate the mitogen-activated protein kinase (MAPK) pathway, contributing to tumorigenesis [30], particularly in cases of PTC with HT. Unlike the more common RET mutations found in medullary thyroid carcinoma (MTC) and MEN2, the most prevalent pro-carcinogenic variant of the RET gene in papillary thyroid carcinoma (PTC) is RET rearrangement [31]. This occurrence is complex and has been the subject of scientific research in recent years. Rhoden et al. [32] detected RET rearrangement in both non-tumor follicular cells of Hashimoto's thyroiditis (HT) and PTC, suggesting a potential shared molecular mechanism between the two. Anna [15] et al. concluded that while RET rearrangement has been reported in PTC, it is a relatively rare event in HT. It has been proposed that HT may induce RET rearrangement, essentially turning it into a gene for thyroid cancer, due to the body's immune response to thyroid cancer antigens. The pathology of HT is characterized by the gradual replacement of thyroid gland epithelial cells with mononucleated cells, leading to suggestions that HT may be considered a precancerous lesion of PTC [33]. In addition, RET rearrangement can activate the downstream signaling system, leading to increased inflammation and creating a microenvironment that promotes tumorigenesis. The HT group in this study had a lower BRAF mutation rate and a higher RET rearrangement rate. The recurrence rate of PTC patients with combined HT was 4.55%, which was lower than the NHT group (9.27%) and the 8%− 28% reported in previous clinical studies [34]. Although the difference in recurrence rate was not statistically significant, further subgroup analysis revealed that the rate of LNM and recurrence was higher in RET-positive patients in the HT group (*P* < 0.05). One-way cox regression analysis suggested that RET rearrangement positivity and its interaction with Hashimoto's effect may be potential risk factors for recurrence. However, after multifactorial regression analysis, only ETE and risk stratification for recurrence were retained as independent predictors of recurrence. Therefore, the role of RET rearrangement in recurrence needs to be further explored in future studies.

The present study has several limitations that should be noted. Firstly, while NGS allows for a more comprehensive detection of genes, the overall sample size is limited and is derived from a single center. Additionally, this study is retrospective in nature, which may be limited by the completeness and accuracy of the clinical case records. Furthermore, the sample population is restricted due to the single center, which could be addressed by expanding the sample size, conducting a multicenter prospective study, and incorporating more imaging data.

In summary, several indicators, including multifocal tumors, TgAb levels, younger age, and negative lymph nodes, can aid in the preoperative diagnosis of HT-combined PTC. Additionally, BRAF mutations are less common in HT-combined PTC and do not significantly impact recurrence. However, the presence of RET rearrangement is associated with a higher recurrence rate in HT-combined PTC, highlighting the importance for clinicians to carefully consider the extent of cleared lymph nodes and resected glands during surgery and to closely monitor patients postoperatively.

## Data Availability

No datasets were generated or analysed during the current study.

## References

[1] Dailey ME, Lindsay S, Skahen R. Relation of thyroid neoplasms to Hashimoto disease of the thyroid gland. AMA Arch Surg. 1955;70(2):291–7. 10.1001/archsurg.1955.01270080137023. 13227748 10.1001/archsurg.1955.01270080137023

[2] Dobrinja C, Makovac P, Pastoricchio M, et al. Coexistence of chronic lymphocytic thyroiditis and papillary thyroid carcinoma. Impact on presentation, management, and outcome. Int J Surg. 2016;28(Suppl 1):S70–4. 10.1016/j.ijsu.2015.12.059. 26708864 10.1016/j.ijsu.2015.12.059

[3] Graceffa G, Patrone R, Vieni S, et al. Association between Hashimoto’s thyroiditis and papillary thyroid carcinoma: a retrospective analysis of 305 patients. BMC Endocr Disord. 2019;19(Suppl 1):26. 10.1186/s12902-019-0351-x. Published 2019 May 29. 31142293 10.1186/s12902-019-0351-x PMC6541562

[4] Zhou L, Chen G, Sheng L, et al. Influence factors for lymph node metastasis in papillary thyroid carcinoma: Hashimoto’s thyroiditis has a weak effect on central or lateral lymph node metastasis. Cancer Manag Res. 2021;13:3953–61. 10.2147/CMAR.S310773. Published 2021 May 14. 34017198 10.2147/CMAR.S310773 PMC8131014

[5] Chen RX, Chen WZ. Research progress on the correlation between Hashimoto’s thyroiditis and papillary thyroid carcinoma. Tumor Prevent Treat. 2022;35(02):186–93.

[6] Ehlers M, Schott M. Hashimoto’s thyroiditis and papillary thyroid cancer: are they immunologically linked? Trends Endocrinol Metab. 2014;25(12):656–64. 10.1016/j.tem.2014.09.001. 25306886 10.1016/j.tem.2014.09.001

[7] Jankovic B, Le KT, Hershman JM. Clinical review: Hashimoto’s thyroiditis and papillary thyroid carcinoma: is there a correlation? J Clin Endocrinol Metab. 2013;98(2):474–82. 10.1210/jc.2012-2978. 23293329 10.1210/jc.2012-2978

[8] Xu S, Huang H, Qian J, et al. Prevalence of Hashimoto thyroiditis in adults with papillary thyroid cancer and its association with cancer recurrence and outcomes. JAMA Netw Open. 2021;4(7):e2118526. 10.1001/jamanetworkopen.2021.18526. Published 2021 Jul 1. 34313737 10.1001/jamanetworkopen.2021.18526 PMC8317012

[9] Hua SR, Liao Q. Parathyroid gland and neuroprotection in postoperative recurrence and reoperation of thyroid cancer. Chinese J Pract Surg. 2021;41(08):871–4. 10.19538/j.cjps.issn1005-2208.2021.08.08.

[10] Zhang Y, Dai JQ, Wu TT, et al. The study of the coexistence of Hashimoto’s thyroiditis with papillary thyroid carcinoma. J Cancer Res Clin Oncol. 2014;140(6):1021–6. 24619663 10.1007/s00432-014-1629-z PMC11823965

[11] Song EY, Jeon MJ, Park S, et al. Influence of coexistent Hashimoto’s thyroiditis on the extent of cervical lymph node dissection and prognosis in papillary thyroid carcinoma. Clin Endocrinol. 2018;88(1):123–8. 10.1111/cen.13475 28906015

[12] Kim GR, Shin JH, Hahn SY, et al. Ultrasonographic features and clinical characteristics of Warthin-like variant of papillary thyroid carcinoma. Endocr J. 2016;63(4):329–35. 26806192 10.1507/endocrj.EJ15-0620

[13] Zhu F, Shen YB, Li FQ, et al. The effects of Hashimoto thyroiditis on lymph node metastases in unifocal and multifocal papillary thyroid carcinoma: a retrospective Chinese cohort study. Medicine. 2016;95(6):e2674. 26871795 10.1097/MD.0000000000002674 PMC4753890

[14] Marotta V, Guerra A, Zatelli MC, et al. BRAF mutation positive papillary thyroid carcinoma is less advanced when Hashimoto’s thyroiditis lymphocytic infiltration is present. Clin Endocrinol (Oxf). 2013;79(5):733–8. 10.1111/cen.12194. 23469895 10.1111/cen.12194

[15] Cyniak-Magierska A, Wojciechowska-Durczyńska K, Krawczyk-Rusiecka K, Zygmunt A, Lewiński A. Assessment of RET/PTC1 and RET/PTC3 rearrangements in fine-needle aspiration biopsy specimens collected from patients with Hashimoto’s thyroiditis. Thyroid Res. 2011;4(1):5. 10.1186/1756-6614-4-5. Published 2011 Jan 10. 21219595 10.1186/1756-6614-4-5 PMC3023781

[16] Mantilla WA, Sanabria-Salas MC, Baldion AM, Sua LF, Gonzalez DM, Lema M. NGS in lung, breast, and unknown primary cancer in colombia: a multidisciplinary consensus on challenges and opportunities. JCO Glob Oncol. 2021;7:1012–23. 10.1200/GO.21.00046. 34185572 10.1200/GO.21.00046 PMC8457807

[17] Wang JQ. Progress in diagnosis and treatment of differentiated thyroid cancer. J Pract Med. 2019;35(20):3258–63.

[18] Hu XR, Xie CP, Chen SY, et al. Clinical characteristics and risk factors of recurrent/persistent differentiated thyroid cancer. Chinese Med Sci. 2021;11(11):12–5.

[19] Kim HJ, Sohn SY, Jang HW, Kim SW, Chung JH. Multifocality, but not bilaterality, is a predictor of disease recurrence/persistence of papillary thyroid carcinoma. World J Surg. 2013;37(2):376–84. 23135422 10.1007/s00268-012-1835-2

[20] Wu K, Shi L, Wang J, Xie L. Association between papillary thyroid carcinoma and lymphocytic thyroiditis: a retrospective study. Oncol Lett. 2023;25(4):148. 10.3892/ol.2023.13734. Published 2023 Mar 1. 36936026 10.3892/ol.2023.13734 PMC10018234

[21] Xu J, Ding K, Mu L, et al. Hashimoto’s thyroiditis: a “Double-Edged Sword” in thyroid carcinoma. Front Endocrinol (Lausanne). 2022;13: 801925. 10.3389/fendo.2022.801925. . Published 2022 Feb 24. 35282434 10.3389/fendo.2022.801925 PMC8907134

[22] Yang Y, Liu J, Shi X, Wang M. Clinical and pathological characteristics of patients with papillary thyroid carcinoma coexisting with hashimoto’s thyroiditis: a retrospective cohort study. Cancer Control. 2023;30: 10732748231199647. 10.1177/10732748231199647. 37643366 10.1177/10732748231199647 PMC10467246

[23] Liang J, Zeng W, Fang F, et al. Clinical analysis of Hashimoto thyroiditis coexistent with papillary thyroid cancer in 1392 patients. Analisi clinica dell’associazione fra tiroidite di Hashimoto e carcinoma papillare della tiroide in 1392 pazienti. Acta Otorhinolaryngol Ital. 2017;37(5):393–400. 10.14639/0392-100X-1709. 29165434 10.14639/0392-100X-1709 PMC5720867

[24] Zhu F, Shen YB, Li FQ, Fang Y, Hu L, Wu YJ. The effects of hashimoto thyroiditis on lymph node metastases in unifocal and multifocal papillary thyroid carcinoma: a retrospective chinese cohort study. Medicine (Baltimore). 2016;95(6): e2674. 10.1097/MD.0000000000002674. 26871795 10.1097/MD.0000000000002674 PMC4753890

[25] Wen X, Wang B, Jin Q, Zhang W, Qiu M. Thyroid antibody status is associated with central lymph node metastases in papillary thyroid carcinoma patients with Hashimoto’s thyroiditis. Ann Surg Oncol. 2019;26(6):1751–8. 10.1245/s10434-019-07256-4. 30937662 10.1245/s10434-019-07256-4

[26] Duan CC, Shen KY, Xiao SQ, et al. Characteristic analysis of cervical lymph node metastasis in papillary thyroid carcinoma combined with Hashimoto’s thyroiditis: literature review. Chinese J Gen Surg Basic Clin Med. 2023;30(12):1507–13.

[27] Eldien MMS, Abdou AG, Rageh T, Abdelrazek E, Elkholy E. Immunohistochemical expression of ER-α and PR in papillary thyroid carcinoma. Ecancermedicalscience. 2017;11: 748. 10.3332/ecancer.2017.748. Published 2017 Jun 13. 28717394 10.3332/ecancer.2017.748 PMC5493440

[28] Zhao W, He L, Zhu J, Su A. A nomogram model based on the preoperative clinical characteristics of papillary thyroid carcinoma with Hashimoto’s thyroiditis to predict central lymph node metastasis. Clin Endocrinol (Oxf). 2021;94(2):310–21. 10.1111/cen.14302. 32984984 10.1111/cen.14302

[29] Janicki L, Patel A, Jendrzejewski J, Hellmann A. Prevalence and impact of BRAF mutation in patients with concomitant papillary thyroid carcinoma and Hashimoto’s thyroiditis: a systematic review with meta-analysis. Front Endocrinol (Lausanne). 2023;14: 1273498. 10.3389/fendo.2023.1273498. Published 2023 Nov 17. 38047109 10.3389/fendo.2023.1273498 PMC10691376

[30] Staubitz JI, Schad A, Springer E, et al. Novel rearrangements involving the RET gene in papillary thyroid carcinoma. Cancer Genet. 2019;230:13–20. 10.1016/j.cancergen.2018.11.002. 30466862 10.1016/j.cancergen.2018.11.002

[31] Subbiah V, Yang D, Velcheti V, Drilon A, Meric-Bernstam F. State-of-the-Art strategies for targeting RET-dependent cancers. J Clin Oncol. 2020;38(11):1209–21. 10.1200/JCO.19.02551. 32083997 10.1200/JCO.19.02551 PMC7145587

[32] Pufnock JS, Rothstein JL. Oncoprotein signaling mediates tumor-specific inflammation and enhances tumor progression. J Immunol. 2009;182(9):5498–506. 10.4049/jimmunol.0801284. 19380798 10.4049/jimmunol.0801284

[33] Deng C, Li S, Yang ZX, et al. Multi-gene assay and clinical characteristics research in papillary thyroid carcinoma. Gland Surg. 2021;10(1):242–51. 33633980 10.21037/gs-20-589 PMC7882307

[34] Sun JH, Li YR, Chang KH, et al. Evaluation of recurrence risk in patients with papillary thyroid cancer through tumor-node-metastasis staging: a single-center observational study in Taiwan. Biomed J. 2022;45(6):923–30. 10.1016/j.bj.2021.11.009. 34808423 10.1016/j.bj.2021.11.009 PMC9795347

